# Academic discourse on education during the early part of the pandemic

**DOI:** 10.1016/j.heliyon.2022.e11170

**Published:** 2022-10-19

**Authors:** Ralph Meulenbroeks, Martijn Reijerkerk, Elisabeth Angerer, Toine Pieters, Arthur Bakker

**Affiliations:** Freudenthal Institute, Utrecht University, Princetonplein 5, 3584 CC Utrecht, the Netherlands

**Keywords:** Higher education, Pandemic, COVID-19, Pedagogy of care, Online education, Blended learning

## Abstract

As the global COVID-19 pandemic forced a sudden transition to emergency online education in early 2020, academic discourse quickly shifted to focus on the new situation and what could be learned from it. The present study gives an overview of the discourse on education during the pandemic in publications that appeared in the top-50 journals on the Clarivate Education list in the period April 2020–May 2021. Based on a final selection of 63 articles and 12 editorials, mostly on higher education, five main themes were identified: affect, teaching practice, teaching context, achievement and assessment, and equity. The academic discourse in these publications indicates that the emergency situation exacerbated previously existing issues: mental distress was observed to rise sharply for all stakeholders and gaps in access to education between different social groups widened. In response, teachers revisited the core values of education to guide them in approaching online teaching. Management focused less on procedures and communicated in a more human and empathic way. We argue that the acute interconnectedness experienced during the pandemic can be used to develop a pedagogy of care in which support is explicitly organized on both socio-emotional and academic levels.

## 1 Introduction

In early 2020, the global educational community was thrown into an unprecedented crisis. Although the severity of the COVID-19 restrictions varied by country and region, students and teachers worldwide were forced to abandon any face-to-face, synchronous, and offline elements of education basically from one day to the next (Engelbrecht et al., 2020; Williamson et al., 2020). From that moment onwards, educational institutions were forced to implement frequently-changing regulations. Parents and caregivers were confronted with the challenge of combining work, care, and education at home. Thus, education entered into what is now referred to as emergency online education or EOE (Williamson et al., 2020).

Partly or fully online education was, of course, not new by this point. Education in general is based on students, teachers, content, and interactions between the three (Bernard et al., 2009; Garrison, 1989). The assumption is often made that if one of the student-interactions (student-student, student-teacher, or student-content) is of a high quality, deep learning will occur, even if the other two are significantly reduced or even minimized (Anderson and Garrison, 1998; Garrison and Vaughan, 2008; Meulenbroeks, 2020; Stein and Graham, 2020). For instance, by focusing almost completely on student-content interaction, Massive Open Online Courses (MOOCs) have demonstrated the theoretical possibility to move education fully online. MOOCs have, however, been consistently plagued by high dropout rates (Li and Moore, 2018; Rivard, 2013), indicating the difficulties with engaging students in fully online education and hinting at the necessity of high-quality teacher and student interaction. In the blended learning approach (Garrison and Vaughan, 2008) a large portion—up to 90%—of all interactions takes place online as well. Importantly, however, blended learning incorporates some form of offline learning and thus, necessarily, synchronous interaction in physical meetings. Blended learning thus attempts to strike a balance between almost unlimited student autonomy in terms of place and time for student-content interaction and the best offline education has to offer in terms of high-quality student-student and student-teacher interactions (Meulenbroeks, 2020; Stein and Graham, 2020). Indeed, many universities have embraced this concept with satisfying results (Bernard et al., 2014).

In the EOE situation, however, all teachers in secondary and tertiary education were forced to abandon *any* offline elements. Most schools and institutions opted for an almost instantaneous transition to fully online education with both synchronous and asynchronous education taking place online, using the ubiquitous videoconferencing tools (Bakker et al., 2021; Bakker and Wagner, 2020; Ellis et al., 2020; Meulenbroeks, 2020).

The educational research community studied the new situation with an eye to possible lessons to learn for improving education. Peer-reviewed publications and editorials started to appear early in the pandemic, addressing the ways that students and teachers adapted to EOE, with issues such as the effectiveness of online education, student and teacher motivation, stress-induced symptoms, assessment, and equity all coming to the fore (Ellaway et al., 2020; Williamson et al., 2020). It was recognized early on that many lessons could be learned from this global education 'experiment'. Indeed, the idea was to *‘…*take advantage of the enforced suspension of most activities to set out the inventory of those among them we would like to see not coming back, and those, on the other hand, that we would like to see develop*’* (Ellis et al., 2020, p. 570).

What is currently missing in the literature is an integrative overview of the diverse publications on the influence of the pandemic on education and stakeholders’ responses to these changes. In this study, we therefore aim to overview the academic discourse on the transition and adaptation to fully online education. The term “discourse” is thus used relatively loosely here, encompassing content in terms of conclusions, implications, and recommendations of research articles and editorials. Focusing on high-impact, peer-reviewed literature based on data collected during the pandemic and editorials published in these journals during the same time, we aim to answer the following research question:

What are the main findings and implications relating to secondary and higher education during the pandemic as presented by high-impact education research journals?

We deliberately designed our research question to be broad in scope, since we aim to be as open as possible towards emerging issues in this unique and unprecedented era in education. In doing so, we intentionally refrain from applying a specific theoretical lens or formulating a priori hypotheses and thus take a bottom-up approach.

## 2 Methods

### 2.1 Selection criteria

To arrive at a sample of articles that was small enough to manage yet broad enough to provide the necessary overview, we decided to narrow our scope on the level of source rather than content. This was done by focussing exclusively on the top-50 journals in the Clarivate Education list, supplemented with two special COVID-19 issues from the top-100 Clarivate Education list (European Journal of Teacher Education and Journal of Computer Assisted Learning). The two top-100 special issues were included because of their large number of articles relating to the subject.

To arrive at our selection the top-50 journals were searched *manually*, using the search engine of the journal itself, with the broad search term: ‘COVID-19 OR pandemic’. The time window for publication was set between February 1, 2020 and May 10, 2021. This manual search resulted in a total of 321 articles. It is possible that some publications may have used different expressions to refer to the virus or the pandemic. Indeed, authors may have chosen to use ‘Corona’ instead of ‘COVID-19’ when referring to the virus. However, since the sample is taken from rigorously peer-reviewed scientific literature, it is considered unlikely that a publication specifically addressing education during the pandemic would not at least also use the more scientific term ‘COVID-19’.

**Figure 1:**
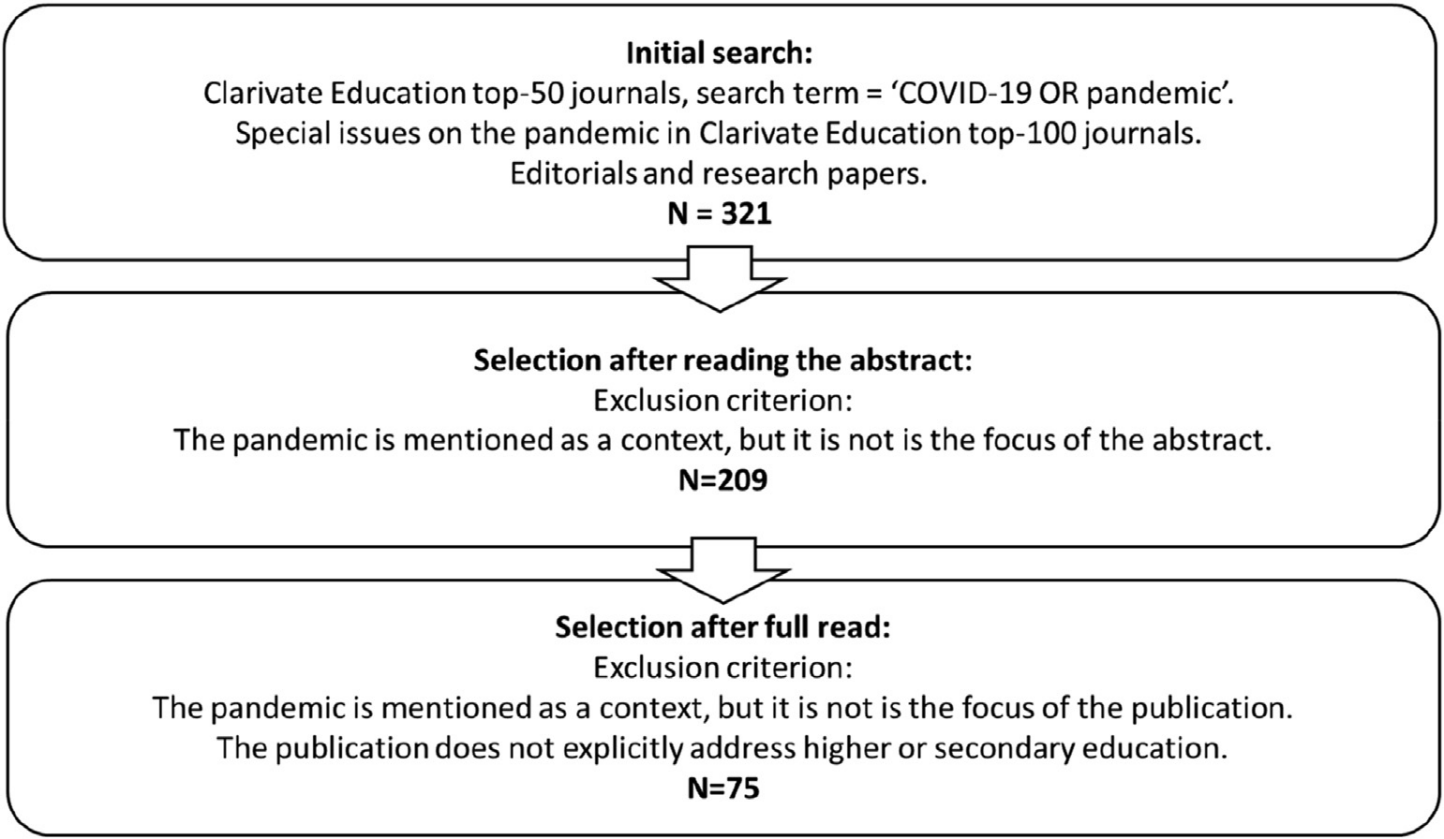
The sample selection process.

Articles in which the abstracts did mention the pandemic but did not specifically address issues directly related to the pandemic or work with data collected during the pandemic were discarded in the first selection step (see Figure 1). This resulted in a sample of 209 articles that were read in full. No publications were discarded based on their specific content or perspective; the only criterium for retaining an article was explicit relation to the pandemic. The exclusion criterion therefore was that the publication (1) did not use data collected during the pandemic or (2) the pandemic was mentioned in passing, but not the focus of the publication. The latter criterion was often used in the selection of editorials. The final selection included 75 publications: 63 research papers and 12 editorials from 16 Top-50 journals and two special issues in Top-100 journals.

### 2.2 The sample

**Figure 2:**
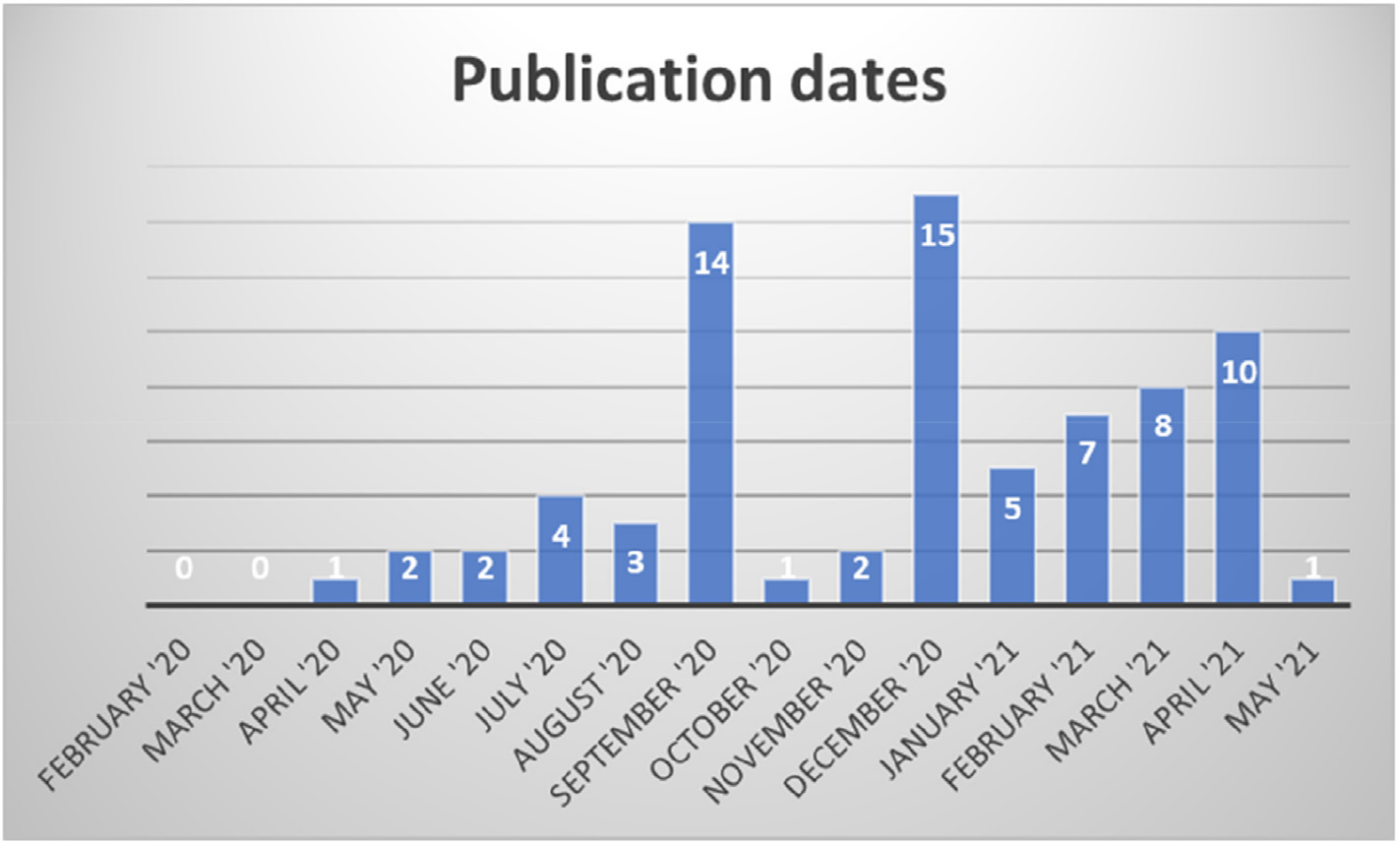
Publication dates of the 75 publications under consideration. The peaks in September 2020 and December 2020 are due to the publication of two special issues on the subject.

The articles in our sample were published in the period from April 2020 through May 10, 2021. Figure 2 visualizes the distribution of the articles over the publication dates, noting that the peaks arise due to the publication of the special issues. Considering both the time lag due to the academic peer-review process and what we could determine from the content, most studies were concerned with the first lockdown in the first half of 2020.

Of the articles, 49 pertain to higher education and 26 to secondary education.

**Figure 3:**
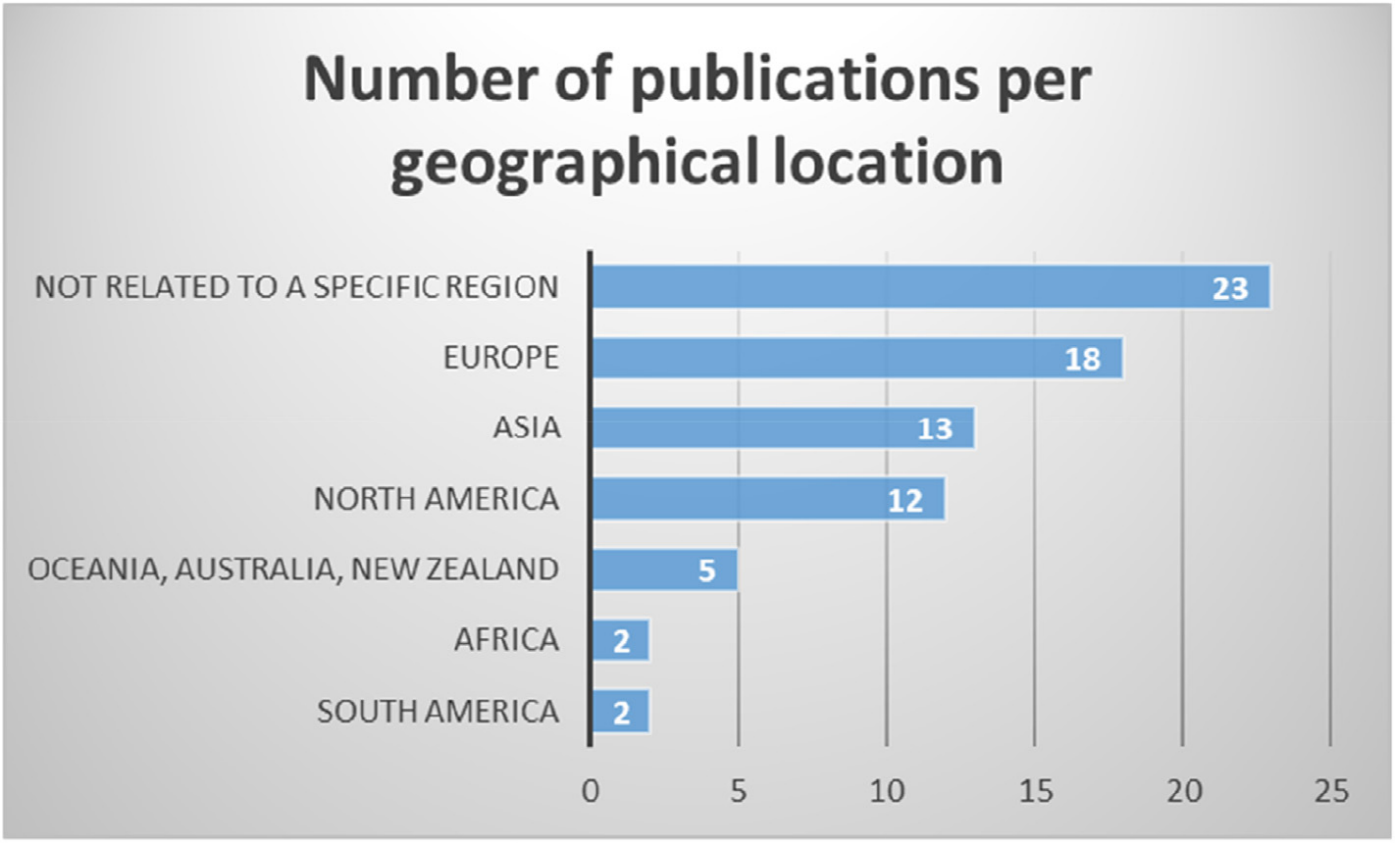
Number of publications per geographical location.

With the inclusion of these special issues the sample has a considerable geographical reach, exemplified by Figure 3.

### 2.3 Theme selection

The findings and implications in the selected publications were studied individually by the authors and organized into overarching themes during five, on-campus, white board discussions with all authors present. Discussions were lengthy and in-depth, taking up to 3 h each. In categorizing the results, the themes identified in a study on mathematics education (Bakker et al., 2021) were taken as a starting point. These themes were: teaching approaches, goals of education, professional development of teachers, technology, equity, affect, assessment, and mathematics education research. Since the present study does not focus solely on mathematics education, however, the decision was made by the authors to slightly alter, merge, or extend these themes.

During the discussions it was decided not to label ‘technology’ as a separate theme: all publications referred to the technological advances and tools that made the transition to fully online education possible, but in the present sample there were no publications focusing on technological issues specifically. Furthermore, ’teaching approaches’ was combined with ‘goals of education’ and renamed ‘teaching practice’. ‘Professional development of teachers’ was extended to include issues at institutional level to become a broader theme of ‘context of teaching’. ‘Assessment’ was extended to also include ‘achievement’ and renamed accordingly. Finally, ‘mathematics education research’ was broadened into ‘education research’ and incorporated into the theme ‘context of teaching’. At the end of the discussion sessions the authors thus reached agreement on five remaining themes: affect, teaching practice, context of teaching, academic achievement and assessment, and equity. Some of the articles and editorials were found to address more than one of the themes and were accordingly assigned to multiple themes. The resulting structure was put into writing by the lead author, reviewed by the co-authors, and discussed during subsequent discussions until full agreement was reached.

## 3 Results

The five themes emerging from the sample of publications were:

(1) Affect (38 publications): elements referring to internal states of teachers, students, managers, parents, and caregivers. For example: motivation, engagement, distress, and stress-related symptoms. Support systems for these stakeholders that were put into place during the pandemic were also included here.
(2) Teaching practice (23 publications): elements relating directly to the teaching process, e.g., online pedagogy and general or online teaching values. Note, however, that assessment is considered together with achievement, see below.
(3) Context of teaching (37 publications): elements relating to the context within which the teaching is taking place, e.g., the universities, schools, or other organizational structures.
(4) Academic achievement and assessment (16 publications): elements relating to the actual performance of students and the forms of assessment being used.
(5) Equity (16 publications): elements relating to diversity and vulnerable student groups.

The results are presented in the form of one overview per theme.

### 3.1 Affect

Table 1 (in three parts) summarizes the findings on this theme, which can be divided into five categories: mental distress as experienced by all stakeholders (students, teachers, parents, and management), support systems for these stakeholders, positive aspects of the corona measures in terms of affect, and gender differences.

**Table 1:** The findings on the theme of Affect: mental distress.

Affect *(38 out of 75 publications)*			
**Widespread mental distress:**			
**Students**	Widespread deteriorating mental health, loneliness, disembodiment, loss of community, depression, insecurity.	- Eringfeld (2021) - König et al. (2020)	- Santibañez and Guarino (2021) - van Schalkwyk (2021)
	Postgraduate students sometimes prefer online education and show greater self-regulation and resilience against external disturbances.	- Tang et al. (2021)	- Yu (2021)
**Teachers**	Deteriorating mental health.	- Hadar et al. (2020) - Moja (2021)	- VanLeeuwen et al. (2021) - Watermeyer et al. (2021)
	Lack of direct interaction with colleagues.	- de Boer (2021) - VanLeeuwen et al. (2021)	- Goedegebuure and Meek (2021)
	A mediating role is played by previous experience with online education.	- van der Spoel et al. (2020)	
	Anxiety about the future of the schooling system as a whole.	- Ellis et al. (2020) - Kidd and Murray (2020)	- O'Brien et al. (2020) - Varea and González-Calvo (2020)
**Parents**	Higher stress levels, balancing home schooling with their own working from home amidst a frightening situation.	- Betts (2021) - Davis et al. (2021)	- Richmond et al., 2020 - Williamson et al. (2020)
**Management**	Stress caused by soaring responsibilities in dealing with constantly changing regulations in combination with their own working from home.	- Beauchamp et al. (2021)	- Thornton (2021)
**Support systems for stakeholders:**			
**Students**	A *pedagogy of care* is proposed: support of students’ socio-emotional needs as well as their academic needs.	- Bebbington (2021) - Darling-Hammond and Hyler (2020) - Ellaway et al. (2020) - Gamage (2021) - Göksu et al. (2021)	- Gravett and Ajjawi (2021) - Kuhfeld et al. (2020) - Santibañez and Guarino (2021) - van Schalkwyk (2021) - Yu (2021)
	Focus on formative assessment, socio-emotional learning, and trauma-informed and healing-informed practices.	- Darling-Hammond and Hyler (2020)	- Hadar et al. (2020)
	Online extra-curricular activities.	- Bebbington (2021)	- Yang and Huang (2021)
**Teachers**	A pedagogy of care is to be incorporated in teacher training programs.	- Carter Andrews et al. (2021)	-
	Teachers should be prepared for an extended duration of the crisis.	- Davis et al. (2021)	- VanLeeuwen et al. (2021)
	A physically safe working environment needs to be provided.	- van der Spoel et al. (2020)	-
	Teacher support in the form of: (a) clear and timely regulations, assessment criteria, and communication; (b) high-quality educational support, including ICT support; (c) training in stress and crisis management methods such as mindfulness; (d) socio-emotional support, e.g., in the form of (online) counselling or a ‘mental health day’ off.	- Hadar et al. (2020) - Murray et al. (2020) - Pressley (2021)	- Truzoli et al. (2021) - VanLeeuwen et al. (2021) - Zhao et al. (2021)
	Support in dealing with critical parents in secondary education.	- Pressley (2021)	
**Parents**	Support for the role of proxy educators and frequent check-ins by a school member are proposed, combined with psychological support.	- Davis et al. (2021)	
	Home and community settings are to be used as ‘reservoirs of knowledge’ for both teachers and teacher educators, also after the pandemic.	- Richmond et al., 2020	
**Management**	Not mentioned in the sample of publications.		
**Positive aspects of the corona measures**	Emerging mutual interdependence between teachers and students but also between teachers and school leaders, a positive feeling of ‘we’re in this together’, e.g., students helping teachers in dealing with ICT difficulties.	- Beardsley et al. (2021)	- Cutri et al. (2020)
	Emerging new teaching styles, sometimes co-designed by teachers and students, e.g., nursing students using virtual reality, co-creation of simulation apps, use of videoconferencing in drama classes.	- Moja (2021)	
	Belonging is redefined as no longer related to a city, building, or time but still requiring active participation of all involved.	- Gravett and Ajjawi (2021)	
	The crisis opens up a possible road towards more caring, human, collaborative, and equal professional relationships.	- Carrillo and Flores (2020)	
	Examples of good practices, e.g., Digital Informal Learning (DIL), with students getting together to discuss educational content at times that have not been scheduled by the school or university. DIL has been demonstrated to enhance students’ engagement in a course, provided they have the necessary digital skills.	- Heidari et al. (2021)	
**Gender differences**	In secondary education female students are reported to lean towards a more positive experience of online teaching than their male counterparts. In higher education, however, no gender differences in terms of affect were reported.	- Tang et al. (2021) - Pressley (2021)	- Truzoli et al. (2021) - Yu (2021)

### 3.2 Teaching practice

The actual teaching practice was deeply influenced in the EOE situation, as is exemplified by the summary of the results in Table 2 (in two parts), which includes the challenges of moving education online, the importance of general teaching values and synchronous activities, as well as the fact that educators became more and more proficient in adapting to the online situation.

**Table 2:** The impact of EOE on the practice of teaching.

Teaching Practice *(21 out of 75 publications)*			
**Online education is more than moving education online**	During the pandemic the continuity of education was facilitated by online education.	- Agasisti and Soncin (2021)	
	Initially, existing classroom practices were just moved online.	- Kidd and Murray (2020)	- O'Brien et al. (2020)
	Teachers quickly took the lead in improving their online pedagogical skills. Virtual teacher training schools were established.	- Almusawi et al. (2021) - Beardsley et al. (2021) - Beauchamp et al. (2021)	- Ellis et al. (2020) - Varea et al. (2020)
**Translating general teaching values to the new situation**	Innovation during the pandemic was based on previous teaching values.	- Kidd and Murray (2020)	- van der Spoel et al. (2020)
	Teachers with a high score on standardized tests related to subject pedagogy before the crisis were more successful in making the transition to online teaching during the crisis.	- König et al. (2020)	
	General teaching values are equal for online and offline education and should include: • accommodation of individual learning styles; • fostering engagement; • providing adequate scaffolding; • fostering student collaboration; • clarifying relevance of and encourage application of theories; • flexibility.	- Carrillo and Flores (2020)	- Kidd and Murray (2020)
	Flipping the classroom can be effectively translated to online education.	- Hew et al. (2020)	
**Synchronous activities are essential**	Synchronous educational activities are indispensable to accommodate student-teacher interaction, even though interaction is less rapid and effective online.	- Truzoli et al. (2021)	
	Hybrid education was shown to exhibit a lower student attendance than fully online, synchronous education.	- Göksu et al. (2021)	
	In general, education should include a live, physical and thus synchronous component. Blended learning is proposed as a viable model for the period after the pandemic.	- Bebbington (2021) - Eringfeld (2021) - Goedegebuure and Meek (2021) - König et al. (2020) -	- Norman (2020) - van der Spoel et al. (2020) - van Schalkwyk (2021) Yang and Huang (2021)
	A complete return to pre-pandemic educational practices is not preferred by students in higher education.	- Eringfeld (2021)	
	In teacher education the perceived value of online teaching increased, but an important offline classroom component was considered essential.	- Ellis et al. (2020)	
**From discomfort to agility**	Once teacher confidence in ICT-skills had developed, technology became a focus of practice, new possibilities, and innovation.	- Kidd and Murray (2020) - Komljenovic (2020)	Sepulveda-Escobar and Morrison (2020)

### 3.3 Context of teaching

All formal teaching takes place within the context of an educational institution. At this organizational level, the existing issues of teacher workload, access to education for vulnerable groups of students, hesitancy towards accepting digital education, and sometimes even distrust towards policy and regulations appear to have become more acute during the crisis (Dvir and Schatz-Oppenheimer, 2020; Watermeyer et al., 2021; Williamson et al., 2020). Many articles in our sample (35 out of 75) addressed the context of teaching. Table 3 (in two parts) gives an overview of the results in terms of support systems, leniency in regulations, emotional leadership, and educational research.

**Table 3:** The results on the context of teaching.

Context of teaching *(35 out of 75 publications)*			
**Supporting (pre-service) teachers and students in using online technology**	Teacher education programs are proposed to provide pre-service teachers with adequate training in digital pedagogy, including online pedagogy for physical education.	- Almusawi et al. (2021) - Carrillo and Flores (2020) - Carter Andrews et al. (2021) - Dvir and Schatz-Oppenheimer (2020)	- O'Brien et al. (2020) - Varea and González-Calvo (2020) - Zhao et al. (2021)
	This support should also address teachers’ ability to transfer the digital skills to their future students, since their students need to adapt to online education as well.	- König et al. (2020)	- Williamson et al. (2020)
	Support for university teachers was provided by online manuals and collections of best practices on how to implement online teaching	- Perrotta (2021)	
	Professional development programs for teachers focusing on digital pedagogy to improve self-efficacy are advocated, in the form of online workshops, lectures, webinars and MOOC’s, and do-it-yourself toolkits.	- Bebbington (2021) - Bragg et al. (2021) - Boltz et al. (2021) - Carter Andrews et al. (2021) - Cukurova et al. (2021) - Darling-Hammond and Hyler (2020)	- Jung et al. (2021) - Moja (2021) - O'Brien et al. (2020) - Tamrat (2020) - Thornton (2021)
	Social media have been successfully employed to support online professional development.	- Beardsley et al. (2021)	- Greenhow et al. (2021)
	The emergence of new and effective online professional development networks is described as one of the positive aspects of EOE	- van der Spoel et al. (2020)	
**Support for vulnerable student groups**	Students from parents with low Social Economic Status (SES), students with disabilities, and international students were labelled as ‘vulnerable’ in the sample under consideration. Proposed supportive measures for these students include grants and subsidies, scholarships, free book programs, and free internet access.	- Coates et al. (2021) - Gurantz and Wielga (2021) - Kuhfeld et al. (2020) - Perrotta (2021)	- Rahman et al. (2021) -Richmond et al., 2020a, Richmond et al., 2020b - Yang and Huang (2021)
	Vulnerable students warrant that their situations and access to internet and devices be closely monitored, especially during prolonged periods of online education.	- Johnson et al. (2021)	
**Leniency in regulations**	Educational institutions at all levels became more lenient in their interpretation of regulations, standards, and procedures: • Large-scale standardized testing—including final secondary school exams—was suspended; • Exceptions on entrance, promotion, and graduation requirement were formulated; • Replacement assignments were offered for practical education; • Curricula were redesigned.	- Chang-Bacon (2021)	de Boer (2021)
	Expectations quickly became more realistic as exceptions to the rules became more common.	- Thornton (2021)	
	Higher education institutions tended to consider the situation more from a student perspective.	- Bebbington (2021)	Eringfeld (2021)
**Emotional leadership and more horizontal organizations**	The crisis required timely translation and implementation of government regulations into local procedures and regulations.	- Agasisti and Soncin (2021) - Bush (2021)	Thornton (2021)
	Educational leadership is reported to have been more emotionally sensitive in nature, expressed in a more empathic, more personal and supportive tone in their communications.	- Beauchamp et al. (2021) - O'Brien et al. (2020)	Thornton (2021)
	The interdependence in a time of crisis resulted in organizations becoming flatter and more open, with less emphasis on hierarchy.	- Ellis et al. (2020) - O'Brien et al. (2020)	Sepulveda-Escobar and Morrison (2020)
	A new kind of distributed leadership as well as a new sense of community appeared, expected to last through the post-pandemic period.	- Eringfeld (2021)	Thornton (2021)
**Educational research**	Proposed questions for further research include subjects such as: • The choreography of student-teacher-content interaction in online education; • Assessment of students’ psycho-social needs; • Virtual learning and its impact on stressors for all stakeholders; • The effectiveness of certain technological tools in education and ways to improve students’ digital competencies; • The application of existing and new theories to elucidate mechanisms in online teaching; • The effects of interrupted schooling in general; • The potential hazards of the growing importance of commercial online learning platforms.	- Benson et al. (2020) - Chang-Bacon (2021) - Cleland et al. (2020) - DeMatthews et al. (2020)	- Ellaway et al. (2020) - Perrotta (2021) - Wang et al. (2021) Williamson et al. (2020)

### 3.4 Academic achievement and assessment

Within our sample, 16 publications specifically addressed the outcomes of education during the pandemic in terms of academic achievement, as well as the way it was assessed in a situation without offline, proctored exams. Table 4 gives a summary of the findings.

**Table 4:** A summary of the results in terms of academic assessment and achievement.

Achievement and assessment*(16 out of 75 publications)*			
**Academic achievement**	Models show that young (primary and secondary) learners in the truncated 2019–20 school year may start the next year with results below 70% of what they would be in a normal year, with mathematics performance at an even lower 50%.	- Kuhfeld et al. (2020)	- Norman (2020)
	With adequate digital pedagogy some students in higher education actually performed better in online situations.	- van Schalkwyk (2021)	- Zhao et al. (2021)
	Academic performance during EOE is linked to support of students’ psychological needs.	- Göksu et al. (2021)	- Santibañez and Guarino (2021)
	In one study, students were best able to reach their higher education learning goals without any digital technology. The use of social media in the educational process is advised against.	- Lacka et al. (2021)	
**Assessment**	After initial hesitations, online testing was widely adopted as a key element in EOE.	- Butler-Henderson and Crawford (2020) - Cutri et al. (2020)	- Truzoli et al. (2021) - Komljenovic (2020)
	Educational institutions at all levels changed their assessment criteria, postponed high-stakes summative testing, extended assessment deadlines, formulated replacement assignments, and changed over to pass/fail assessments instead of grades.	- de Boer (2021) - Jung et al. (2021)	- König et al. (2020) - Moja (2021)
	In higher education, students were reported to prefer online assessment to offline assessment once they got used to it.	- Butler-Henderson and Crawford (2020)	
	Teachers considered giving formative feedback during EOE much more time-consuming than offline. Their confidence in providing feedback online increased once they got used to it.	- Beardsley et al. (2021)	

### 3.5 Equity

The one conclusion which all authors addressing the subject agreed upon was that the pandemic and EOE severely exacerbated existing gaps between different societal groups. Access to online education does not only imply access to the internet and devices (however essential) but also includes a safe and at least relatively quiet working space for students and a supportive environment. Both issues (access and environment) are addressed by publications in our sample (16 out of 75). Table 5 summarizes the findings the widening of the digital divide, ways to mitigate it and signs of hope from the pandemic.

**Table 5:** A summary of the results as related to equity.

Equity *(16 out of 75 publications)*			
**The digital divide was exacerbated by EOE**	Reasons for the widening of the gap: poorer access to internet, devices, and appropriate working spaces for students from a low SES background, students with disabilities, international students, or students in unstable home and living conditions.	- Benson et al. (2020) - Boltz et al. (2021) - Cutri et al. (2020) - Gamage (2021) - Göksu et al. (2021) - Gurantz and Wielga (2021) - Kidd and Murray (2020) - Kuhfeld et al. (2020)	- Moja, 202 - O'Brien et al. (2020) - Rahman et al. (2021) - Santibañez and Guarino (2021) - Unterhalter and Howell (2021) - van Schalkwyk (2021) - Watermeyer et al. (2021)
	Some authors expressed their fears for a widening of the gender gap in academia.	- Jung et al. (2021)	
**Mitigating the gap**	Schools and institutions can compensate for unfavorable home situations by offering: • Free internet access; • Affordable laptop schemes; • Adequate learning spaces at schools or universities; • Better ICT integration at schools or institutions.	- Göksu et al. (2021) - González-Betancor et al. (2021) - Johnson et al. (2021)	- Perrotta (2021) - Williamson et al. (2020)
	Some authors advocated for adapting teacher training in order to make pre-service teachers more aware of the digital divide and ways to spot and mitigate it.	- Flores and Swennen (2020)	- Murray et al. (2020)
**Hope**	Online education carries with it the possibility for educational institutions to become more open, accessible, affordable, democratic, and socially embedded.	- Eringfeld (2021) - Gamage (2021)	- Perrotta (2021)
	Further research into the effects of availability and use of internet and devices in online education is needed.	- Gourlay et al. (2021)	- Williamson et al. (2020)

### 3.6 Answering the research question

We now revisit our research question:

What are the main findings and implications relating to secondary and higher education during the pandemic as presented by high-impact education research journals?

As an important starting note, many studies noted that overall, the COVID-19 crisis *exacerbated existing issues* rather than created new ones. This was true for all five themes that were addressed above. For example, a survey among UK university faculty staff reports distrust towards prolonged and institutionalized digital pedagogies, in line with pre-pandemic studies on this issue (Watermeyer et al., 2021). Respondents of the same study referred to how their existing difficulties in finding a proper work-life balance were exacerbated by the increased workload during the crisis. Likewise, also in secondary education existing issues such as work pressure, work-life balance, and hesitancy towards the digitalization of education appeared to have been experienced more acutely during the pandemic (Williamson et al., 2020). Indeed, EOE… *‘…*brought important issues to the forefront that we could ignore in previous times, particularly those related to inequalities in access to education*’* (Benson et al., 2020, p. 351).

The articles and editorials we examined paint a stark picture of an entire sector that was forced to demonstrate an unprecedented level of flexibility during the pandemic. Recent developments in videoconferencing technology and other web-based tools enabled this flexibility. Graduate students had less trouble adapting to the online situations, including online assessments, than their undergraduate and sub-graduate counterparts. But the transition always came at a cost.

This was more often than not a cost in human terms. As mentioned above, many underlying issues in education were amplified by the transition to EOE. Teachers, students, management, and parents/caregivers almost universally—and globally—reported sharply increases in levels of mental distress during the pandemic, resulting in higher levels of teacher burnout, student loneliness, fear, dehumanization, and work pressure in general. Existing procedures that used to rely on some form of physical presence (e.g., internships, practical assignments, or assessment) had to be abandoned, adapted, or loosened by educational organizations. Parents and caregivers needed to juggle their multiple responsibilities even more than before the pandemic. Issues of inequity in access to education—in its broadest sense—were also sharpened, as students from lower SES backgrounds, students with disabilities, or students in developing countries saw their already limited access to education dwindle in the new situation and adequate access to internet and devices and proper workspaces were often unavailable.

Yet during this profoundly unsettling experience, the educational world also learned a lot. Teachers were able to learn from their students on how to work online, and students adjusted to online assessment. The interconnectedness and interdependence of all the diverse stakeholders came to the forefront: We *were* all in it together. The strengthened sense of community opened up new interaction cultures: organizations learned how to communicate more openly and with more empathy, and parents/caregivers learned a lot about education. Mutual support became more common for all stakeholders, taking the form of technical and pedagogical support in terms of webinars and do-it-yourself manuals for teachers and students, but also psychological support via online sessions or the ubiquitous social media. For future practice, the concept of blended learning with synchronous online as well as offline meetings, is widely advocated by the authors in our sample.

Due to the mostly lengthy peer-review process the articles in our sample relate to a relatively short period of time in the early pandemic or do not specifically address a time frame at all, as in the case of some editorials. Therefore, the material does not readily lend itself for a longitudinal interpretation. Authors do refer to some negative aspects of EOE wearing off as people got used to them, e.g., students getting used to online testing once their anxiety wore off and they got used to the software (Butler-Henderson and Crawford, 2020) and teachers exploring the newly discovered possibilities (Kidd and Murray, 2020).

Two main ideas that may well last beyond the pandemic are offered by the publications. These ideas point the way to addressing deeper issues that were highlighted during the pandemic.

First, several authors referred to what we might call ‘pedagogy of care’, i.e., stakeholders taking care of and caring about each other as a fundamental quality of education. In this pedagogy of care, support is explicitly organized on the socio-emotional as well as the academic levels.

Second, the explicit interdependence which developed during the crisis forced universities and schools to put less emphasis on hierarchy and procedures. This distributed leadership with a more human face, in which administrators attempted to ‘look through the eyes’ of students and teachers, could well become a model to continue after the crisis.

To be flexible, educators were forced to turn to their core values. Values in online education did not appear to differ much from values in more regular education: fostering engagement and collaboration, offering support, placing content in its relevant context, and offering flexibility. During the pandemic, these educational values were observed to be more valuable than ever for educators.

## 4 Discussion

### 4.1 Limitations

Our approach has been somewhat unorthodox, deviating from a formal review procedure as described, for example, in Brereton et al. (2007). Since a wide search in a major search engine with a simple search term of the kind ((COVID-19 OR pandemic) AND education) yields about 23,000 articles since 2020, the search term would need to be very specific in order to arrive at a reasonable number of articles for review. We argued that this would lead to a loss of comprehensiveness, in conflict with our aim of obtaining an impression of the academic discourse on the *whole* issue.

We therefore opted to be very open in the search term (COVID-19 OR pandemic), retaining feasibility and timeliness by restricting the sampling to the top-50 high-impact Clarivate journals on education and two special issues in the Clarivate top-100 on education. In doing so, we aimed to select only articles that had gone through a rigorous peer review process, which we expect will be reflected in a high quality of procedure. This quality would also be reflected in the editorials in these same high-impact journals, or so we argued. We expect that the exclusion of other, more casual, references to the virus (e.g., “Corona”) from the search term will not lead to data loss, since a peer-reviewed publication that is specifically addressing the pandemic is not likely to exclusively use a more casual reference to the virus.

Our approach led to a relatively small number of journals which we were able to consider in depth. As the very nature of the peer-review process is time consuming, the time span between data collection and publication in our sample is relatively long. This implies that the publications in our selection relate to a rather short period of time in the beginning of the pandemic. Furthermore, all journals were in English. On the other hand, we found a manageable, broad, and high-quality sample of 75 articles and editorials with reports from five continents covering a broad area of topics and a variety of disciplines. We therefore feel confident that our approach led to a fairly representative overview of the main issues reported on during the early phase of the pandemic.

### 4.2 Future research

Several areas of research were advocated for in the sample of articles and editorials under consideration. The complex choreography of student-teacher-content interaction in online education merits further study (Cleland et al., 2020), as does the assessment of students’ psycho-social needs (DeMatthews et al., 2020), and the impact of virtual learning on all stakeholders as mentioned in Table 1 (Benson et al., 2020; Ellaway et al., 2020). The use of videoconferencing and other technology comes with its own questions on the effectiveness of technological tools in education and ways to improve students’ digital competencies (Benson et al., 2020; Wang et al., 2021; Williamson et al., 2020). On a more fundamental level, the application of existing and formulation of new theories to elucidate mechanisms in online teaching has been advocated (Ellaway et al., 2020). The effects of interrupted schooling and remediation warrant further study (Chang-Bacon, 2021). Finally, the potential hazards of the growing importance of commercial online learning platforms in relation to more traditional schooling organizations has been suggested as an area of research (Perrotta, 2021).

## 5 Conclusion

The pandemic has taught us that the educational community can be flexible to the extreme, but at a cost in terms of mental suffering for many stakeholders. And yet we cannot exclude the possibility of a crisis like this happening more often in an increasingly globalized and complex world. Disruptions may indeed become part of the new normal (Davis et al., 2021). The present study demonstrates that in order to remain flexible and minimize the negative effects of these disruptions, a focus on core values is essential.

The core values of education, such as fostering engagement and offering scaffolding, inclusiveness, and relevance need to be (and remain) guiding principles, especially when the outside world is changing rapidly and unpredictably. These values are to be the inner compass in times of crisis. An emergency may force certain educational practices and procedures to change, maybe irrevocably so, but it will not change the fundamentals of education. As an illustration, a firm basis in pedagogy was reported to make it easier for teachers to make the transition to fully online education (König et al., 2020).

Online or predominantly online education—with its extended possibilities of access—has an enormous democratizing potential (Perrotta, 2021). Notwithstanding, our study suggests that the period of fully online education has widened, not closed, the digital divide and actual access to education (Benson et al., 2020). Hence, realizing the democratizing potential of online education is by no means automatic.

We as authors express our hope that the results of this study may contribute to an atmosphere of reflection upon the basic human values within education, of taking a conscious step back in the aftermath of the crisis, and contemplating, indeed, the possibility of making *‘…a more decisive set of significant social and digital changes’* (Williamson et al., 2020, p. 111)*.*

We propose to extend a pedagogy of care to the entire educational community. Focusing on the general values of education, especially on equity and ‘looking through the other person’s eyes’, this pedagogy of care is envisaged to become the basis of a more global vision for the future of education. A vision in which academic and socio-emotional needs are on an equal footing, in which values are more important than procedures, in which educators, students, managers, and parents/caregivers are all conscious of their interrelated responsibilities in a global ecosystem, and in which we can work towards fulfilling the basic human right to education.

## Ethical statement

This study complies with the regulations of the ethical board of the Faculty of Science and Geology of Utrecht University. The study involves no human subjects. The authors explicitly deny any conflict of interest in the context of this study.

## Declarations

### Author contribution statement

All authors listed have significantly contributed to the development and the writing of this article.

### Funding statement

This research did not receive any specific grant from funding agencies in the public, commercial, or not-for-profit sectors.

### Data availability statement

Data will be made available on request.

### Declaration of interest’s statement

The authors declare no conflict of interest.

### Additional information

No additional information is available for this paper.
